# A New Diterpene from *Litsea cubeba* Fruits: Structure Elucidation and Capability to Induce Apoptosis in HeLa Cells

**DOI:** 10.3390/molecules19056838

**Published:** 2014-05-23

**Authors:** Piyapat Trisonthi, Akihiko Sato, Hisashi Nishiwaki, Hirotoshi Tamura

**Affiliations:** 1 The United Graduate School of Agricultural Science (UGAS), Ehime University, 3-5-7 Tarumi, Matsuyama, Ehime Prefecture 790-8566, Japan; 2 The Graduate School of Agriculture, Kagawa University, 2393 Ikenobe, Miki, Kagawa Prefecture 761-0795, Japan

**Keywords:** *Litsea cubeba*, cubelin, apoptosis, HeLa cell, caspase

## Abstract

A new diterpene, identified as (+)-6-(4-hydroxy-4-methyl-2-pentenoyl)-4,6-dimethyl-5-(3-methyl-2-butenyl)-1,3-cyclohexadienecarbaldehyde (**1**, cubelin), was isolated from a methanol extract of *Litsea cubeba* fruits by normal phase column chromatography and purified by preparative HPLC. The structure elucidation was conducted by spectroscopic methods (UV, IR, ESI-TOF-MS, 1-D and 2-D NMR). Cubelin exhibited activity against HeLa cell viability and proliferation. The cells also exhibited changes in nuclear morphology which are hallmarks of apoptotic cell death. The presence of cleaved caspase-3/-7, caspase-8 and caspase-9 in the cubelin treated population indicated the potential of the compound to induce apoptosis in HeLa cells via both intrinsic and extrinsic pathways.

## 1. Introduction

*Litsea cubeba* (Lour.) Pers is an evergreen tree or shrub, belonging to the Lauraceae family. *L. cubeba* fruit is rich in essential oil (May Chang oil), which is produced by steam distillation. In China, approximately 1,500–2,000 tons of May Chang oil are produced yearly. The maximum essential oil content of *L. cubeba* fruit is approximately 5.0% of the fresh weight [1,2]. The oil is an economical source of citral for cosmetic and food industries. It is also an aromatherapy component as an anti-depressant agent [1]. May Chang oil has been widely studied for its chemical components, which are mainly monoterpenes and sesquiterpenes. The major chemical constituent of this oil is citral, which comprises geranial (*E*-citral) and neral (*Z*-citral) [3,4,5,6]. The oil has been found to have bio-activities such as anti-inflammatory, antioxidant, pesticidal, antimicrobial, anticancer and neuropharmacological properties [7,8,9,10,11,12]. However, past research on biological active substances in *L. cubeba* fruits have mainly focused on the essential oil, while the possible existence of beneficial non-essential oil compounds has not been explored. Therefore, the objective of our research is to discover some new biological active compounds in *L. cubeba* fruits. In this study we focused on anticancer compounds by evaluating apoptosis-inducing efficiency.

The search for new anticancer agents is an important element for the improvement of cancer treatment strategies. The increased understanding about the relationship between cancer and the cell cycle disruption has led to more attention in research on the cell cycle targeting therapeutic methods. Plant-derived bioactive compounds have been an important material for chemotherapy. Most of the chemotherapeutic drugs are active against cancer cells by restoring the function of cell cycle arrest and apoptosis [13]. However, there are many negative side effects from chemotherapy such as nausea, vomiting, diarrhea, renal toxicity, genotoxic effect and decreased blood cells [14]. Furthermore, cancer cells could develop resistance against chemotherapy agents by several mechanisms [15]. Therefore, continuous research attempts to find more new natural compounds with anticancer activity are still important for the future of cancer treatment.

In the present study, a diterpene compound, identified as (+)-6-(4-hydroxy-4-methyl-2-pentenoyl)-4,6-dimethyl-5-(3-methyl-2-butenyl)-1,3-cyclohexadienecarbaldehyde (**1**, cubelin, C_20_H_28_O_3_), was newly isolated from the methanol extract of *L. cubeba* fruits. This compound induced apoptosis in a human cervical cancer cell line (HeLa) by activation of the apoptotic caspase cascade. The chemical structure is quite unique and it has not been reported, based on a similarity search in the SciFinder and the ChemSpider databases.

## 2. Results and Discussion

### 2.1. Structure Elucidation

Compound **1**, (+)-6-(4-hydroxy-4-methyl-2-pentenoyl)-4,6-dimethyl-5-(3-methyl-2-butenyl)-1,3-cyclohexadienecarbaldehyde, was obtained as a pale yellowish oil with a specific optical rotation [α]20 D of +274.025 (*c* = 0.02, CHCl_3_) and maximum UV absorptions at 216 and 324 nm. The molecular weight minus a hydroxyl group was determined by HRESIMS as C_20_H_28_O_2_ (obsd. at *m/z* 299.2017 [M−OH+H]^+^, cald. *m/z* 299.2011) and another peak was observed as C_20_H_27_O_3_Na^+^ (obsd. at *m/z* 339.1911 [M+Na]^+^, cald. *m/z* 339.1936). The data suggested that the molecular formula of **1** is C_20_H_28_O_3 _(*m/z* 316)_, _which indicated seven degrees of unsaturation. The IR spectrum showed hydroxyl group (3516–3393 cm^−1^, broad), unsaturated carbons (3034 cm^−1^), saturated carbons (2978 cm^−1^) and overlapping bands of conjugated aldehyde and ketone (1670 cm^−1^) absorption bands.

The structure elucidation was explained based on NMR spectroscopic data (Table 1). Chemical shifts (δ) were reported in part per million (ppm), standardized by chemical shift of TMS and residue of CDCl_3_. In total, 20 signals were observed in the ^13^C-NMR spectrum. Signals interpreted by the DEPT experiment suggested the presence of six aliphatic methyls at δ 16.46, 17.85, 24.04, 25.81, 29.26 and 29.47, one aliphatic methylene at δ 25.57, one aliphatic methine at δ 46.27, one aliphatic quaternary carbon at δ 52.99, one tertiary oxygenated carbon at δ 71.20, five olefinic methine carbons at δ 118.94, 121.83, 123.09, 148.45 and 153.04, three tertiary olefinic carbons at δ 132.60, 136.37 and 156.38, one aldehyde carbon at δ 193.06 and one ketone carbon at δ 200.28. These data supported the molecular formula as C_20_H_28_O_3._ The ^1^H-NMR spectrum revealed one aldehyde proton at δ 9.46 (1H, s) and six methyl groups at δ 1.28, 1.30, 1.39, 1.52, 1.62 and 1.95 (3H, s). According to the correlation based on the ^1^H-^1^H COSY spectrum, a methylene group at δ 2.08 (2H, dd, *J* = 6.50, 7.56 Hz) has spin coupling with two adjacent protons which are an aliphatic methine proton at δ 2.73 (1H, t, *J* = 6.50 Hz) and a vinyl methine proton at δ 5.04 (1H, t, *J* = 1.38, 7.56 Hz). This datum indicated the existence of a [C=C_(13)_H-C_(4)_H_2_-C_(8)_H-] fragment. In addition, two vinyl methine protons at δ 6.54 and δ 6.88 (1H, d, *J* = 15.12 Hz), and two methine protons at δ 5.87 and δ 6.83 (1H, d, *J* = 5.52 Hz) were also correlated to be a [-C_(12)_H=C_(17)_H-] fragment, and a [C=C_(11)_H-C_(16)_H=C] fragment, respectively.

**Table 1:** ^1^H and ^13^C-NMR correlation based on HETCOR spectrum for cubelin in CDCl_3_ (*δ*in ppm, *J* in Hz).

Position	δ ^13^C	δ ^1^H	No.	δ ^13^C	δ^ 1^H
1	16.4	1.39 (3H, s)	11	118.9	5.87 (1H, d, *J* = 5.52)
2	17.8	1.52 (3H, s)	12	121.8	6.54 (1H, d, *J* = 15.12)
3	24.0	1.95 (3H, s)	13	123.0	5.04 (1H, t, *J* = 1.38, 7.56)
4	25.5	2.08 (2H, dd, *J* = 6.50, 7.56)	14	132.6	
5	25.8	1.62 (3H, s)	15	136.3	
6	29.2	1.28 (3H, s)	16	148.4	6.83 (1H, d, *J* = 5.52)
7	29.4	1.30 (3H, s)	17	153.0	6.88 (1H, d, *J* = 15.12)
8	46.2	2.73 (1H, t, *J* = 6.50)	18	156.3	
9	52.9		19	193.0	9.46 (1H, s)
10	71.2		20	200.2	

**Figure 1:**
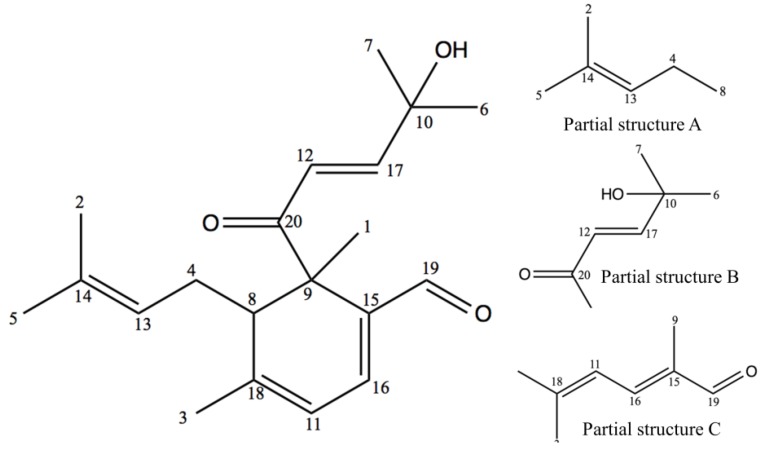
Chemical structure of cubelin (**1**) and its partial structures A, B and C. The numeration was assigned based on the ^13^C-NMR spectroscopic data.

**Figure 2:**
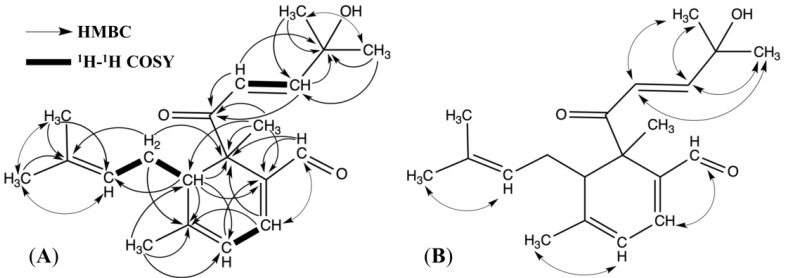
HMBC and ^1^H-^1^H COSY correlations (**A**) and NOESY correlations (**B**) of cubelin.

The chemical structure of cubelin (Figure 1) was constructed based on the long ranged proton-carbon correlations as displayed in the HMBC spectrum (Figure 2A). As for the fragment containing [C=C_(13)_H-C_(4)_H2-C_(8)_H-] unit assigned by *J* coupling with H-13, H-4 and H-8, the correlations between H-13 and C-5, H-5 and C-2, C-14, and H-2 and C-13, C-14 were clearly observed and suggested connections between C-14 and two terminal methyl groups. This data set established the partial structure A (Figure 1). From the [-C_(12)_H=C_(17)_H-] fragment, correlation between H-12 and C-10, C-20, and then between H-17 and C-10, C-20, between H-6 and C-7, C-10, C-17 and between H-7 and C-10, C-17, suggested a connection between C-12 and C-20. C-17 is attached to C-10, which is also connected to two terminal methyl groups. The chemical shift of C-10 (δ 71.20) suggested the presence of a hydroxyl group. These data clearly supported the partial structure B (Figure 1). For the fragment containing the [C=C_(11)_H-C_(16)_H=C] unit, cross-peaks between H-16 and C-9, C-18, C-19, and between H-19 and C-9, C-15, as well as between H-11 and C-15 suggested a connection between C-19 and C-15. Therefore, C-15 should be connected to C-16 and C-9. At the other edge of the fragment, cross-peaks between H-3 and C-11, C-18 suggested that C-11 is connected to C-18 with a methyl group (H-3). This data set established the partial structure C (Figure 1). Finally, the correlation between H-8 and C-9, C-11, C-13, C-15, C-18, C-20 and between H-1 and C-8, C-9, C-15, C-20 enabled construction of the entire chemical structure of cubelin (Figure 1) by connecting all three partial structures, because the connection of C-8 to both C-9 and C-18 suggested the presence of a monocyclic structure. Obvious NOE signals were observed between H-12 and H-6/7, H-17 and H-6/7, H-19 and H-16, H-11 and H-3, H-13 and H-5 (Figure 2B). The present result still does not clarify the configuration at C-8 and C-9, but since two monoterpene units composed by head to tail connection of isoprene units were conjugated at C-8 and C-9; C-15 and C-16, we suppose that both major side chains at C-8 and C-9 might have *trans*-configurations because of the steric hindrance.

**Figure 3:**
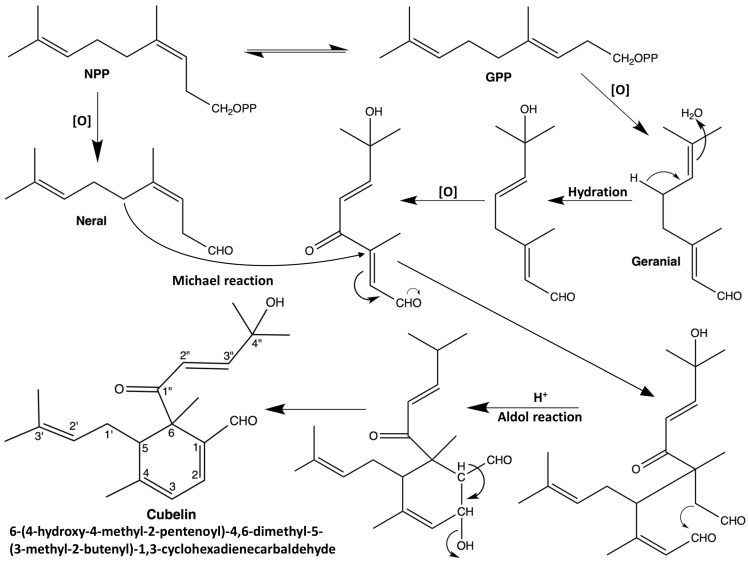
Proposed biosynthesis pathway of cubelin. The numeration in cubelin structure was assigned based on the rule of IUPAC nomenclature.

We have not found any reported chemicals in *L. cubeba* or even in the *Litsea* genus that have a chemical skeleton similar to cubelin. However, partial structure C (Figure 1) is related to that of geranial and neral, which are the major components of *L. cubeba* fruit essential oil. Therefore, we presume that cubelin might be biosynthesized from conjugation of these compounds. A proposed biosynthesis of cubelin is shown in Figure 3.

### 2.2. The Anticancer Potential of Cubelin

Cancer generally occurs as a consequence of mutation in genes that modulate tissue homeostasis. More than half of all human cancers are caused by loss of the tumor suppressor p53 function, which regulates cell cycle arrest and the apoptotic caspase cascades. Mutation of the p53 gene leads to declined apoptotic cell death and unlimited cell proliferation [16,17]. Apoptosis is a biological process that eliminates defected cells without causing inflammatory responses [18]. The major enzymes involved in this process are proteolytic enzymes in caspase family. The effector, caspase-3/-7 regulates apoptosis by cleaving cytoskeleton proteins, nuclear proteins and cell survival relating proteins. Zymogens of the effector caspases must be cleaved by the initiator caspases to convert into their active form. Initiators, caspase-8 and -9 are activated through different pathways, which are the death receptors (extrinsic) and the mitochondrial released cytochrome c (intrinsic) pathways, respectively [19].

In this study, we determined the potential of cubelin to induce apoptosis in the human cervical cancer cell (HeLa). The positive control used in this study was etoposide (VP-16), which is an anticancer drug that promotes apoptosis by inhibition of topoisomerase II [20,21]. Firstly, we investigated effect of cubelin on viability (WST-8 formazan measurement), proliferation (BrdU incorporation) and release of LDH (INT formazan measurement) in the HeLa cell populations that were exposed to various concentrations of cubelin and etopside (1–100 µM) for 24 and 48 h.

According to the results in Table 2, cubelin was effective against HeLa cell viability with an average IC_50_ of 34.43 mM at 24 h and 21.92 at 48 h. The amount of replicating DNA in the cubelin- treated population was also decreased (IC_50_: 30.49 µM at 24 h and 24.08 µM at 48 h). This data suggested that cubelin suppressed HeLa cell proliferation. Leakage of LDH from cytoplasm is an indicator for cell membrane rupture, which occurs as a result of secondary necrosis in the late apoptosis stage *in vitro* [22,23]. It was found that cubelin exhibited mild activity on LDH leakage (IC_50_: 179.21 µM at 24 h and 144.55 µM at 48 h). However, etoposide also exhibited a similar effect with even weaker activity. This result indicated that LDH leakage in the cubelin- and etoposide-treated populations did not occur immediately after the test compounds were administered. Therefore, it could be assumed that cubelin might induce cell death by apoptosis rather than necrosis, which is a type of cell death that rapidly occurs as a result of external stress and causes inflammatory responses that damage the surrounding cells [24].

**Table 2:** Activity of cubelin and etoposide on HeLa cells viability, proliferation and LDH leakage.

Time/Sample		IC_50_ (µM) ^a^		
		Cell viability	LDH leakage	Cell proliferation
24 h	Cubelin	34.43 ± 4.87	179.21 ± 6.23	30.49 ± 4.29
	Etoposide	62.90 ± 5.48	No activity	34.24 ± 5.59
48 h	Cubelin	21.92 ± 3.58	144.55 ± 2.43	24.07 ± 1.55
	Etoposide	4.52 ± 1.02	201.17	3.17 ± 1.96

^a^ IC_50_ values were calculated from dose-response curves after 24 h and 48 h of exposure.

**Figure 4:**
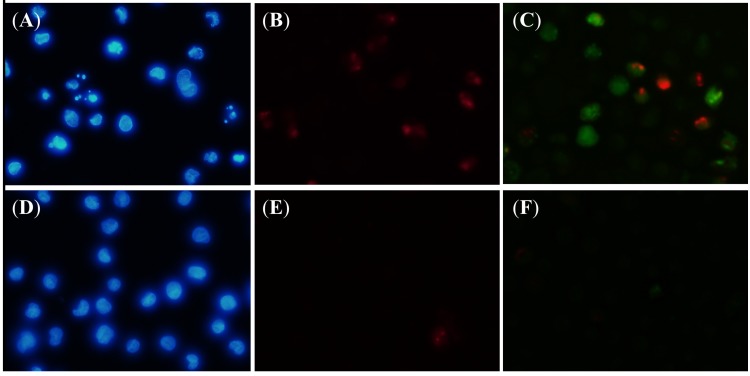
Chromatin condensation pattern in the HeLa cells treated with 50 µM cubelin (**A**) and in the untreated population (**D**) was revealed by using Hoechst 33342. Dead cells in the cubelin treated (**B**) and the untreated population (**E**) were revealed by PI. The presence of caspase-3/-7 was clearly evident in the cubelin treated cells (**C**), while that in the untreated population (**F**) was not obvious.

The cells undergoing apoptosis have specific morphological changes which are: (1) cell membrane blebbing; (2) condensation and margination of DNA and (3) fragmentation of nuclei. Apoptotic bodies are formed after these changes and then they are eliminated by phagocytosis [19,21]. Figure 4 shows a fluorescent microscopic view of HeLa cells that were exposed to 50 µM of cubelin for 24 h, compared with that of the untreated population. Nuclear content were revealed by Hoechst 33342 staining. The cubelin treated cells exhibited morphological characteristics of apoptotic cell. Nuclei breakdown was observed as well as chromatin condensation (Figure 4A), while the untreated cells did not exhibit these characteristics (Figure 4D). Identification of dead cells was done by propidium iodide (PI) staining, which is a nucleotide binding dye that lacks of cell membrane permeability. More PI positive cells were observed in the cubelin treated population (Figure 4B) when compare to the untreated cells (Figure 4E). Figure 4C,F display the cells stained with fluorescent caspase-3/-7 inhibitor (FAM-DEVD-FMK) in the cubelin treated population and the untreated population, respectively. Significant intensity of the dye was observed only in the cubelin treated population. This result is the important evidence that suggest about apoptosis induction potential of cubelin.

**Figure 5:**
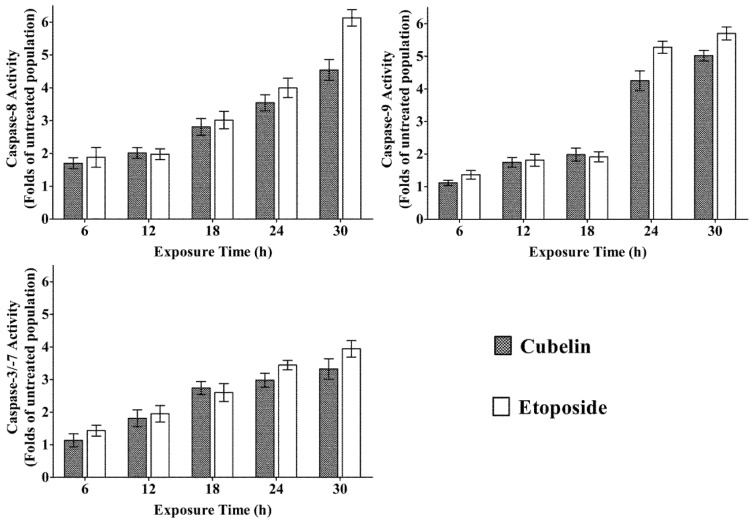
Effect of cubelin and etoposide (50 µM) on induction of HeLa cell apoptosis. Activity of caspase-8 (**A**), caspase-9 (**B**) and caspase-3/-7 (**C**) were measured and compared with that of the untreated population.

The cleaved effector and initiator caspases in the cubelin treated HeLa cell population was measured quantitatively at 6–30 h after administration of cubelin (50 µM) and etoposide (50 µM). The result is presented as folds of the spontaneously cleaved caspases in the untreated population (Figure 5). The amount of cleaved caspase-8 in cubelin treated population gradually increased from 1.7, 2.0, 2.8, 3.5, and 4.5 folds, respectively (Figure 4A). The amount of cleaved caspase-9 did not significantly increase at 6–18 h, but increased sharply to 1.9 folds at 18 h, to 4.2 folds at 24h and then reached 5.0 folds at 30 h (Figure 4B). This result suggested that administration of cubelin had an effect on activation of initiator caspase-8 and -9, which lead to activation of the effector caspase-3/-7 which is the major manipulator of apoptosis. The amount of caspase-3/-7 in the cubelin treated population gradually increased from 1.1, 1.8, 2.7, 2.9 and 3.3 folds of that in the untreated population at 6, 12, 18, 24 and 30 h of exposure, respectively (Figure 4C). These data clearly suggested the potential of cubelin on the caspase cascade in HeLa cells. This compound was found to be effective in promotion of apoptosis via both the death receptor pathway (caspase-8 activation) and the mitochondrial released cytochrome c (caspase-9 activation).

In this study, we found that this new diterpene compound from *L. cubeba* fruit has potential as an anticancer agent. In comparision to etoposide, which is a clinical cancer drug, cubelin exhibited slightly lower activity in promoting caspase cascades (Figure 5). The apoptosis-inducing effect of cubelin is very interesting for further study to investigate the exact anticancer mechanism, such as inhibition of topoisomerase, the effect on function of oncogenes and tumor suppressor genes, and the structure-activity relationship.

Another noteworthy point is that cubelin has a new chemical skeleton. This compound might be useful as a lead compound for further development of more effective anticancer agents.

## 3. Experimental Section

### 3.1. General Experimental Procedures

Optical rotation was recorded on a P-1030 polarimeter (JASCO International, Tokyo, Japan). 1-D NMR spectra (^1^H, ^13^C and DEPT) and 2-D NMR spectra (^1^H-^1^H COSY, HETCOR, HMBC and NOESY) at 600 MHz was recorded in CDCl_3_ with 0.05% TMS as the internal standard, using a JEOL ECA-600 spectrometer (JEOL, Tokyo, Japan). The chemical shifts (δ) are reported in parts per million (ppm) referenced by signals of TMS and residue of CDCl_3_. The HRESIMS spectrum was recorded on a Waters Xevo QTOF-MS spectrometer (Waters Corporation, Milford, MA, USA). The UV-VIS spectrum was recorded on a Shimadzu SPD-M20 Diode Array Detector (Shimadzu Corporation, Kyoto, Japan). The IR spectrum was recorded on a Jasco FT-IR-400 spectrometer.

### 3.2. Plant Material

Fruits of *Litsea cubeba* were harvested in September 2010, from a mountainous area in Mae-Rim district, Chiang Mai, Thailand. The fresh fruits were washed and air dried before shipping to Japan by airmail. The voucher specimen (LC14001TAFS6-1) has been deposited at The Research Institute of Food Safety and Nutraceutical Science, Kagawa University, Japan.

### 3.3. Extraction, Isolation and Identification of (+)-6-(4-Hydroxy-4-methyl-2-pentenoyl)-4,6-dimethyl-5-(3-methyl-2-butenyl)-1,3-cyclohexadienecarbaldehyde (Cubelin, **1**)

Air dried *L. cubeba* fruits (2 kg) were ground into a coarse powder and then extracted with hexane (3× 4L). The remaining fruit residue was extracted again with methanol (3× 4L). After the methanol extract was filtered and the solvent was removed under vacuum, the extract (300 g) was dissolved in diethyl ether (2 L). The diethyl ether soluble filtrate (50 g) was then subjected to silica gel column chromatography (Wakogel C-300, 200 × 50 mm, 45–75 μm particle size) after the solvent was removed. Elution was done with mixtures of hexane and diethyl ether (1:0, 3:1, 1:1, 1:3 and 0:1, 1L each) to give 20 fractions (250 mL/fraction). Fraction 11 and 12, which were eluted by 1:1 mobile phase, were subjected to preparative RP-HPLC (Mightysil RP-18GP, 250 × 10 mm, isocratic: 54% Acetonitrile, 0.1% TFA in H_2_O, flow rate: 0.6 mL/min, UV-VIS detector: 230 nm) to afford cubelin (retention time: 120–125 min). The amount of cubelin obtained from 2 kg of fresh fruit was 64 mg. Pale yellowish oil; [α]20 D +274.025 (*c* = 0.02, CHCl_3_); UV *λ_max_* (logε): 216 (2.99), 324 (2.69) nm; IR (KBr): *υ_max_* 3597, 3516–3393 (broad), 3034, 3010, 2978, 1670, 1629, 1561 cm^−1^; HRESIMS: *m/z* 299.2017 [M−OH+H]^+^ (calcd. 299.2011 for C_20_H_28_O_2_), *m/z* 339.1911 [M+Na]^+^ (cald. *m/z* 339.1936 for C_20_H_28_O_3_Na^+^). ^1^H and ^13^C-NMR data are shown in Table 1.

### 3.4. General Procedure on Cell Culture and Cell Based Assays

The Human Cervical Cancer cell line (HeLa) was purchased from the RIKEN Bioresource Center, (Ibaraki, Japan). The stock culture was grown in EMEM, supplemented with 4.2 mM HEPES, 10% FBS, 0.1 mg/mL penicillin and streptomycin. Incubating condition was set to 37 °C, humidified atmosphere with 5% CO_2_. Exponentially growing cells were harvested and incubated in assay vessels for 24 h prior to sample administration. All test samples were dissolved in DMSO, which final concentration did not exceed 0.1%. Untreated population was exposed to 0.1% DMSO. Treated populations in cell viability, cell proliferation and LDH determination assays were exposed to 1–100 µM of cubelin and etoposide (positive control). Absorbance in colorimetric assays was recorded on a Multiskan™ FC Microplate Photometer. The IC_50_ values were calculated with GraphPad Prism 5.0.

### 3.5. Cell Viability Assay

Cell viability was determined by using a Cell Counting Kit-8 (Dojindo Molecular Technologies, Kumamoto, Japan). In a 96-well transparent cell culture plate, HeLa cells (3 × 10^3^ cells/well) were exposed to the test samples for 24 and 48 h. Ten microliter of CCK-8 reagent was added to each well then the cells were further incubated for 240 min. The absorbance was recorded at 450 nm. Cell viability in each treated population was expressed as viability percentage compared to the untreated population.

### 3.6. Cell Proliferation Assay (BrdU Incorporation)

The cell proliferation assay was carried out by using the cell proliferation ELISA BrdU (colorimetric) kit (Roche Applied Science, Penzberg, Germany). HeLa cells (1 × 10^4^ cells/well) were exposed to the test samples for 24 and 48 h and then labeled with BrdU solution (100 µM) for 120 min under the cell incubating condition. The cells were fixed with a FixDenat solution and then exposed to the Anti-BrdU peroxidase solution for 90 min at room temperature. After washing, tetramethylbenzidine (TMB) was added to reveal the immune complex. H_2_SO_4_ (0.2 M) was used as a stop solution. Absorbance value was recorded at 450 nm and the cell proliferation percentage of each treated population was calculated by comparing to the untreated population.

### 3.7. Determination of LDH Leakage

Determination of LDH was performed with a Cytotoxicity Detection Kit ^plus^ (Roche Applied Science, Penzberg, Germany). EMEM supplemented with 1% FBS was used as an assay medium and two sets of untreated population were assigned. HeLa cell at 5 × 10^3^ cells/well density was exposed to the test samples for 24 and 48 h. One set of untreated population was treated with 5% Triton-X-100 for 15 min, representing maximum LDH leakage in the certain cell population size (high control). The amount of LDH was determined by adding a mixture of tetrazolium salt INT, sodium lactate, NAD^+^ and diaphorase, and incubating for 4 min at room temperature. HCl (1 N) was used as a stop solution. The absorbance was measured at 492 nm. The percentage of leaked LDH was calculated in comparison to the absorbance of high control after subtraction of the absorbance of untreated population.

### 3.8. Determination of Activated Caspase-8, Caspase-9 and Caspase-3/-7

Determination of caspases was carried out using a Caspases Determination Kit―FAM-FLICA (Immunochemistry Technologies, LLC, Bloomington, MN, USA). Caspase substrate FAM-LETD-FMK, FAM-LEHD-FMK and FAM-DEVD-FMK were used for labeling caspase-8, caspase-9 and caspase-3/-7, respectively. HeLa cell suspension (8 × 10^4^ cells/mL) were seeded into 100 mm petridishes and incubated for 24 h. The cells were exposed to cubelin (50 µM), etoposide (50 µM) and DMSO (0.1%) for 6–36 h. Treated cells were harvested by using a trypsin solution (0.25% *w/v*). The cell suspension was spun (400 *×g*) and re-suspended in the culture medium and the cell density was adjusted to 4 × 10^6^ cells/mL. The cell suspensions were separately exposed to each caspase substrates (1:150) for 90 min at 37°C. Excessive substrate was neutralized by washing solution containing mammalian proteins and 0.01% sodium azide. After washing, the cells were re-suspended in PBS and the cell concentration was adjusted to 2.5 × 10^6^ cells/mL. The cell suspension was transferred into black solid 96-well plates (100 µL/well). Relative fluorescent unit (RFU) was measured at 485 nm excitation and 530 nm emission wavelengths on a CYTOFLUOR series 4000 fluorescent plate reader.

### 3.9. Fluorescent Microscope Analysis

FAM-DEVD-FMK and PI dual staining was conducted following the method of caspases determination assay. The cells stained with FAM-DEVD-FMK was exposed to PI (1.25 µg/mL) for 10 min at 37 °C. Hoechst 33342 staining was conducted individually. After re-suspension with culture medium, the cells were exposed to Hoechst 33342 (1 µg/mL) for 10 min at 37 °C. After washing, the cells were re-suspended in PBS and the density was adjusted to 2.5 × 10^6^ cells/mL. The cell suspension (10 µL) was smeared on a glass slide and mounted with the ProLong^®^ Gold antifade reagent (Invitrogen) before being observed under fluorescent microscope (Olympus BX51, Olympus Optical, Tokyo, Japan) at 200× magnification.

## 4. Conclusions

A new cytotoxic diterpenoid compound, cubelin, was isolated from a methanol extract of *L. cubeba* fruits. This compound induced apoptosis in HeLa cells by promoting activation of initiator caspase-8 and -9, resulting in an increased level of the cleaved effector caspase-3/-7. This compound has not been reported as one of the components in *L. cubeba* fruit essential oil. We proposed a hypothesis that cubelin might be biosynthesized from neral and geranial, which are major components of the oil.

## Supplementary Materials

The 1-D and 2-D NMR data, the HRESIMS spectrum, the IR spectrum, the UV-VIS spectrum and the specific optical rotation data are provided as supplementary materials, which can be accessed at: http://www.mdpi.com/1420-3049/19/5/6838/s1.
