# Supplementary material for: A New Diterpene from Litsea cubeba Fruits: Structure Elucidation and Capability to Induce Apoptosis in HeLa Cells
Source: Molecules. 2014 May 23;19(5):6838–50. doi: 10.3390/molecules19056838 (PMC6271781; doi:10.3390/molecules19056838)
Supplement: Supplementary file 1 [file molecules-19-06838-s001.pdf]

Supplementary Materials

Figure S1. IR spectrum of cubelin.

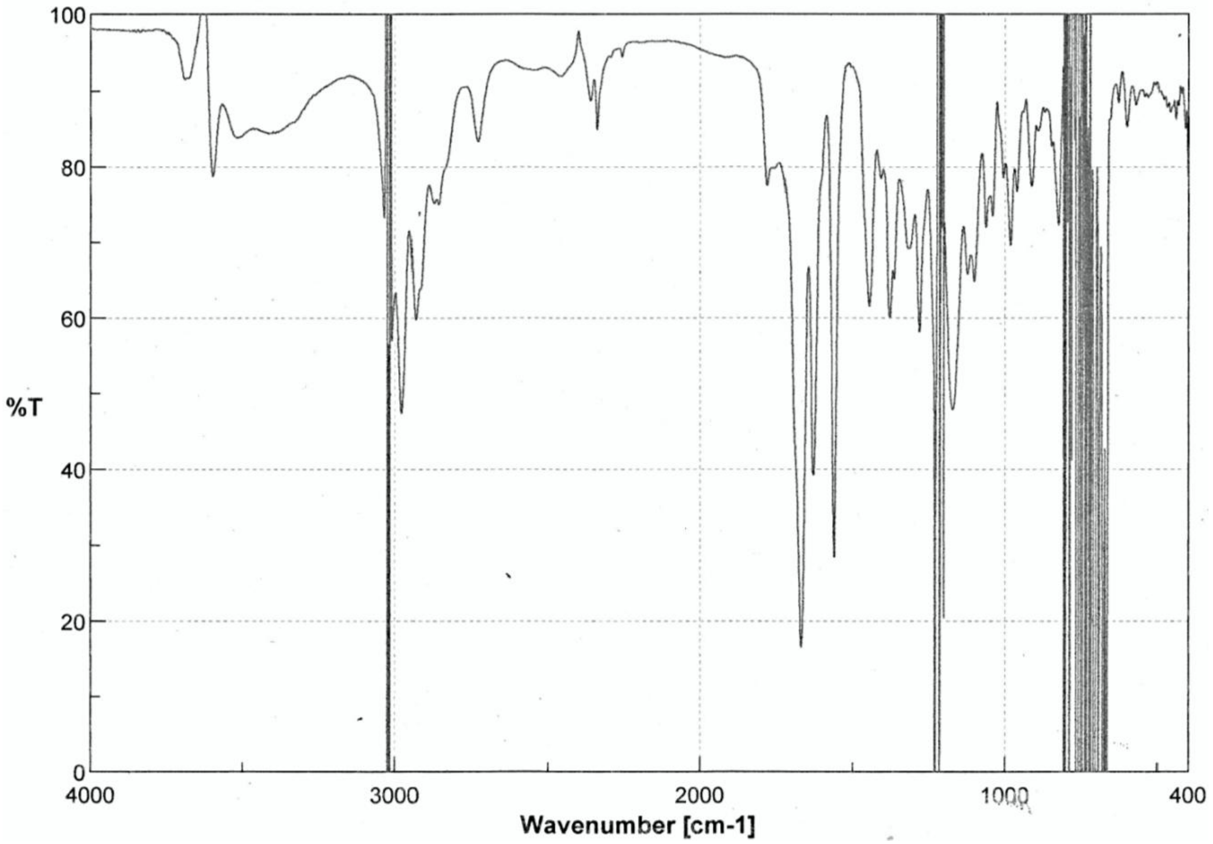

**Accumulation:**	4
**Resolution:**	4 cm^{-1}
**Zero Filling:**	ON
**Apodization**:	Cosine
**Gain:**	Auto (2)
**Aperture:**	Auto (7.1 mm)
**Scan Speed**:	Auto (2 mm/sec)
**User:**	
**Acquisition Date:**	2013/11/16 17:42
**Modify Date:**	2013/11/25 19:31
**File Name:**	C1-4mg.jws
**Sample Name:**	
**Comment:**	

Figure S2. HRESIMS spectrum of cubelin.

Elemental Composition Report **Page 1**

Single Mass Analysis
Tolerance = 50.0 mDa / DBE: min = -1.5, max = 50.0
Element prediction: Off
Number of isotope peaks used for i-FIT = 3

Monoisotopic Mass, Even Electron Ions
56 formula(e) evaluated with 8 results within limits (all results (up to 1000) for each mass)
Elements Used:
C: 0-30 H: 0-1000 O: 0-200
131118_1_2 619 (4.769)
1: TOF MS ES+

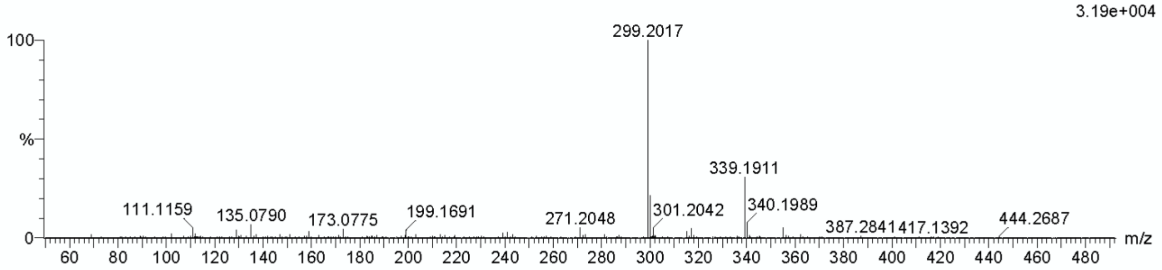

Minimum:				-1.5			
Maximum:		50.0	10.0	50.0			

Mass	Calc. Mass	mDa	PPM	DBE	i-FIT	i-FIT (Norm)	Formula
299.2017	299.2011	0.6	2.0	7.5	159.2	1.6	C20 H27 O2
	299.2070	-5.3	-17.7	-1.5	160.3	2.6	C13 H31 O7
	299.1858	15.9	53.1	3.5	159.6	1.9	C16 H27 O5
	299.2222	-20.5	-68.5	2.5	159.2	1.6	C17 H31 O4
	299.1800	21.7	72.5	12.5	160.7	3.1	C23 H23
	299.1706	31.1	103.9	-0.5	161.1	3.5	C12 H27 O8
	299.2375	-35.8	-119.7	6.5	159.8	2.1	C21 H31 O
	299.1647	37.0	123.7	8.5	159.3	1.7	C19 H23 O3

Figure S3. UV-VIS spectrum of cubelin.

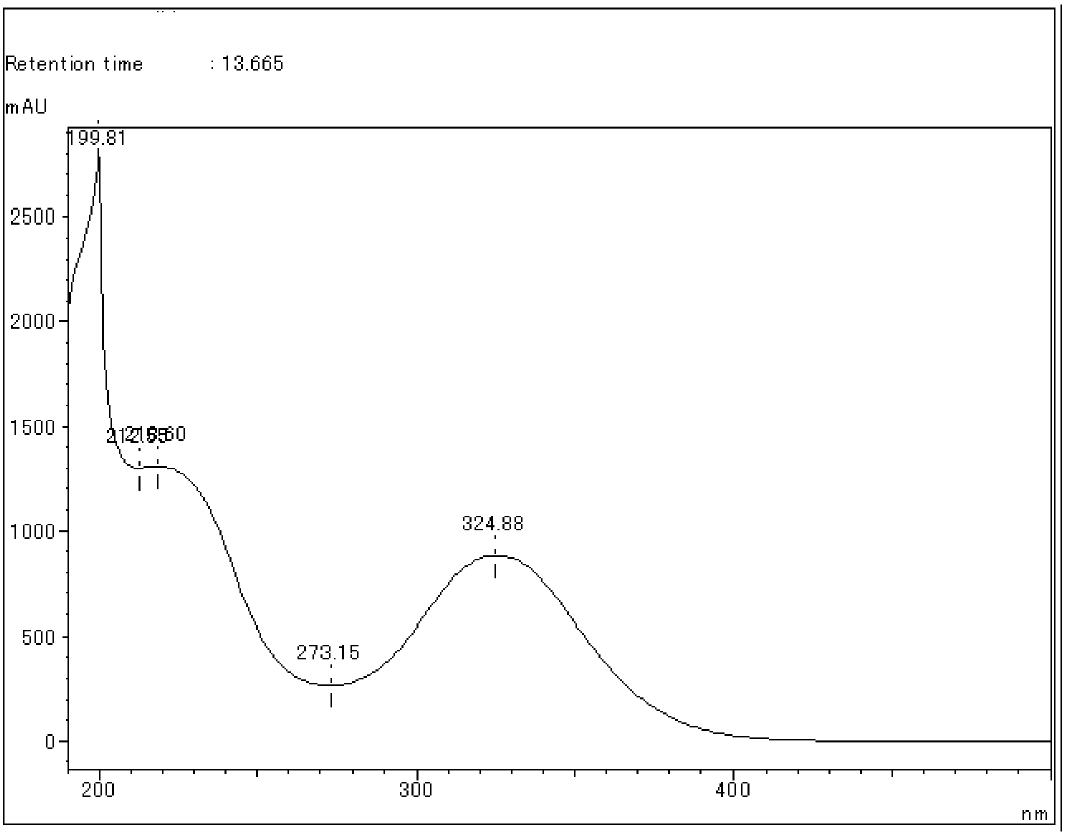

HPLC-DAD Chromatogram: Fraction 11

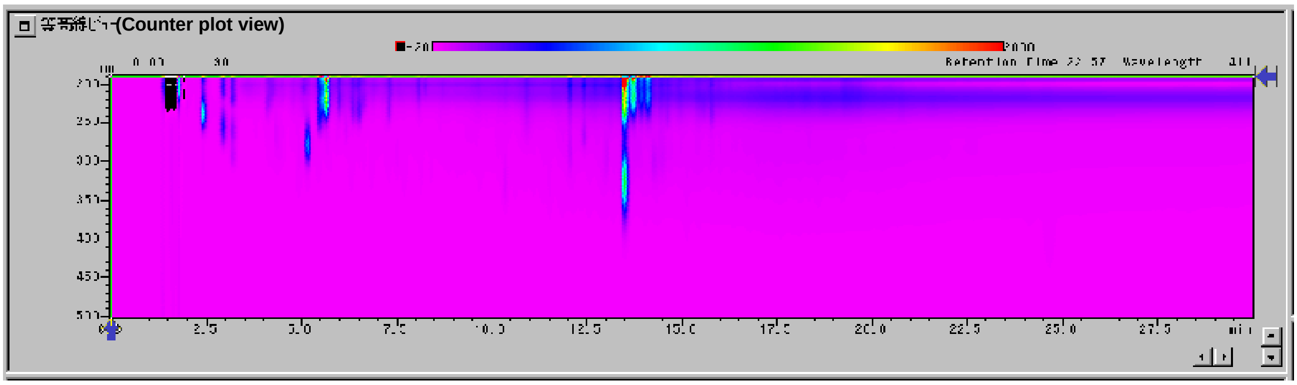

HPLC-DAD Chromatogram: Fraction 12

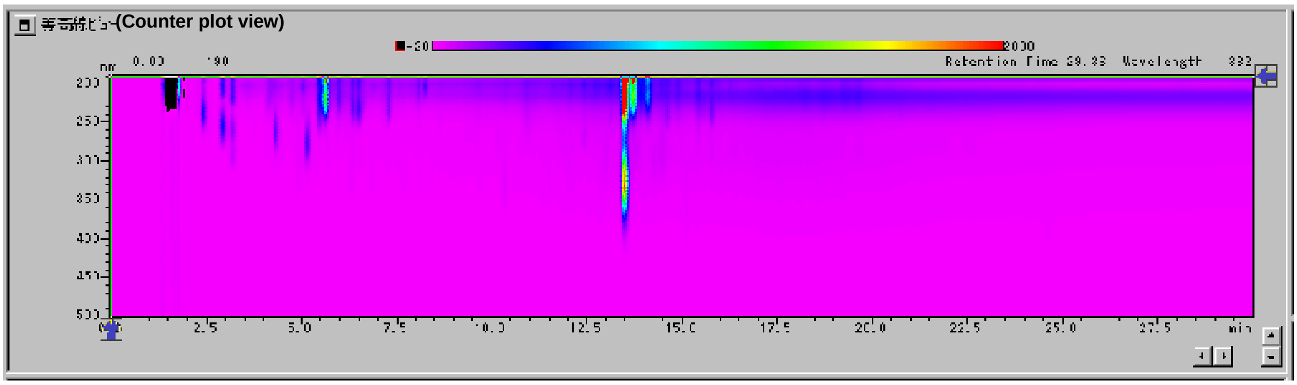

Figure S4. ^{1}H-NMR spectrum of cubelin.

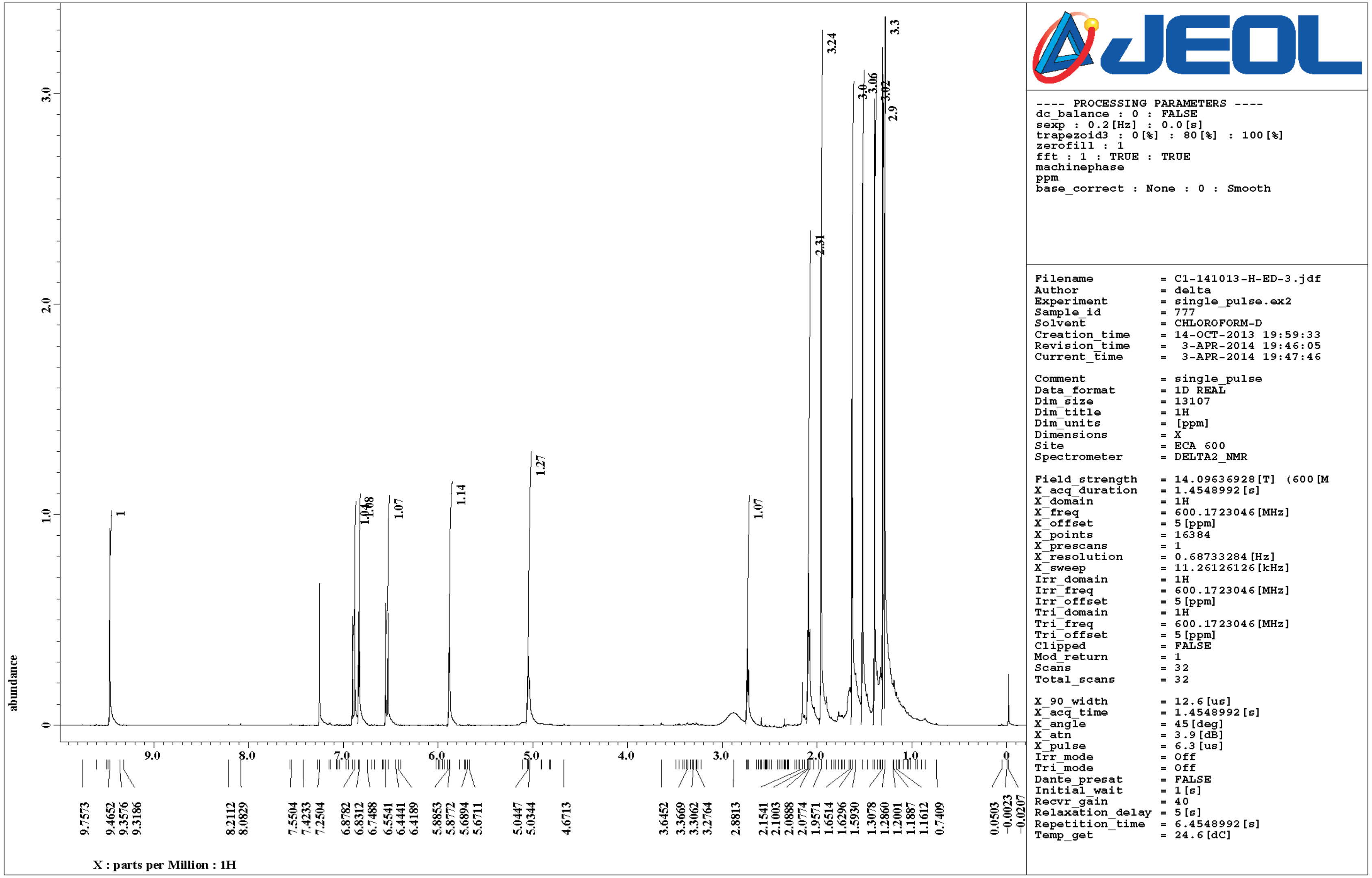

Figure S5. ^{13}C-NMR spectrum of cubelin.

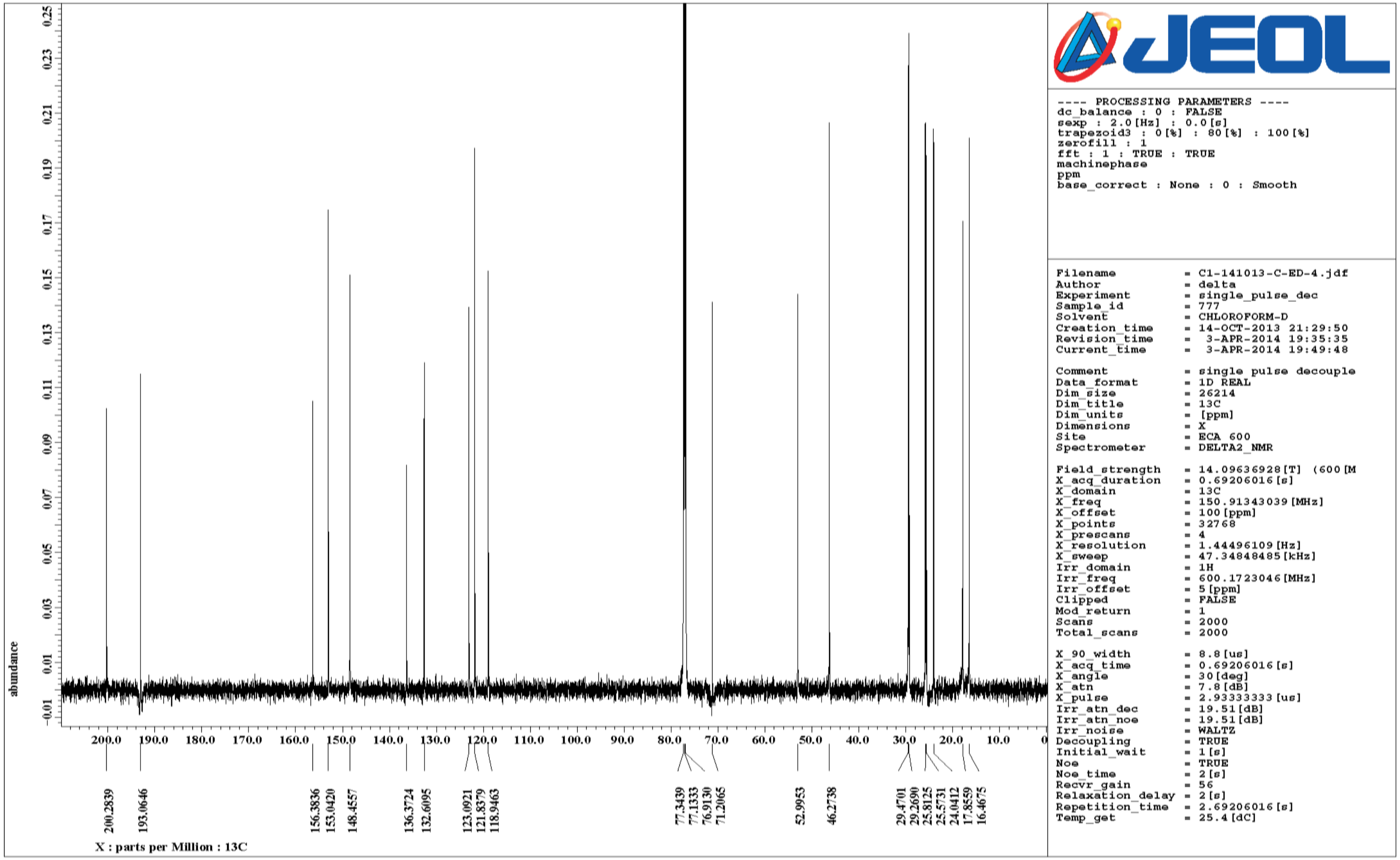

Figure S6. DEPT-45 NMR spectrum of cubelin.

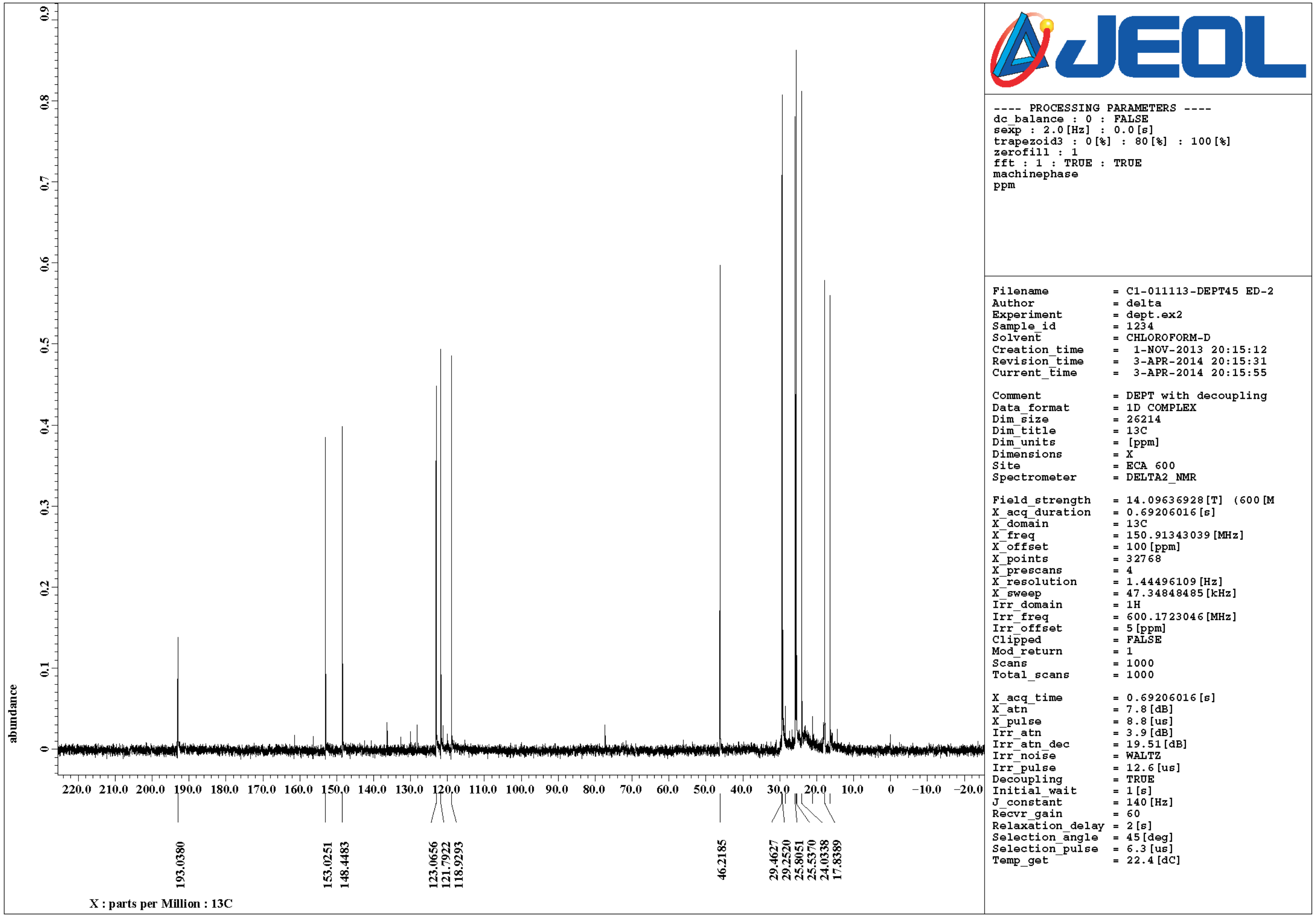

Figure S7. DEPT-90 NMR spectrum of cubelin

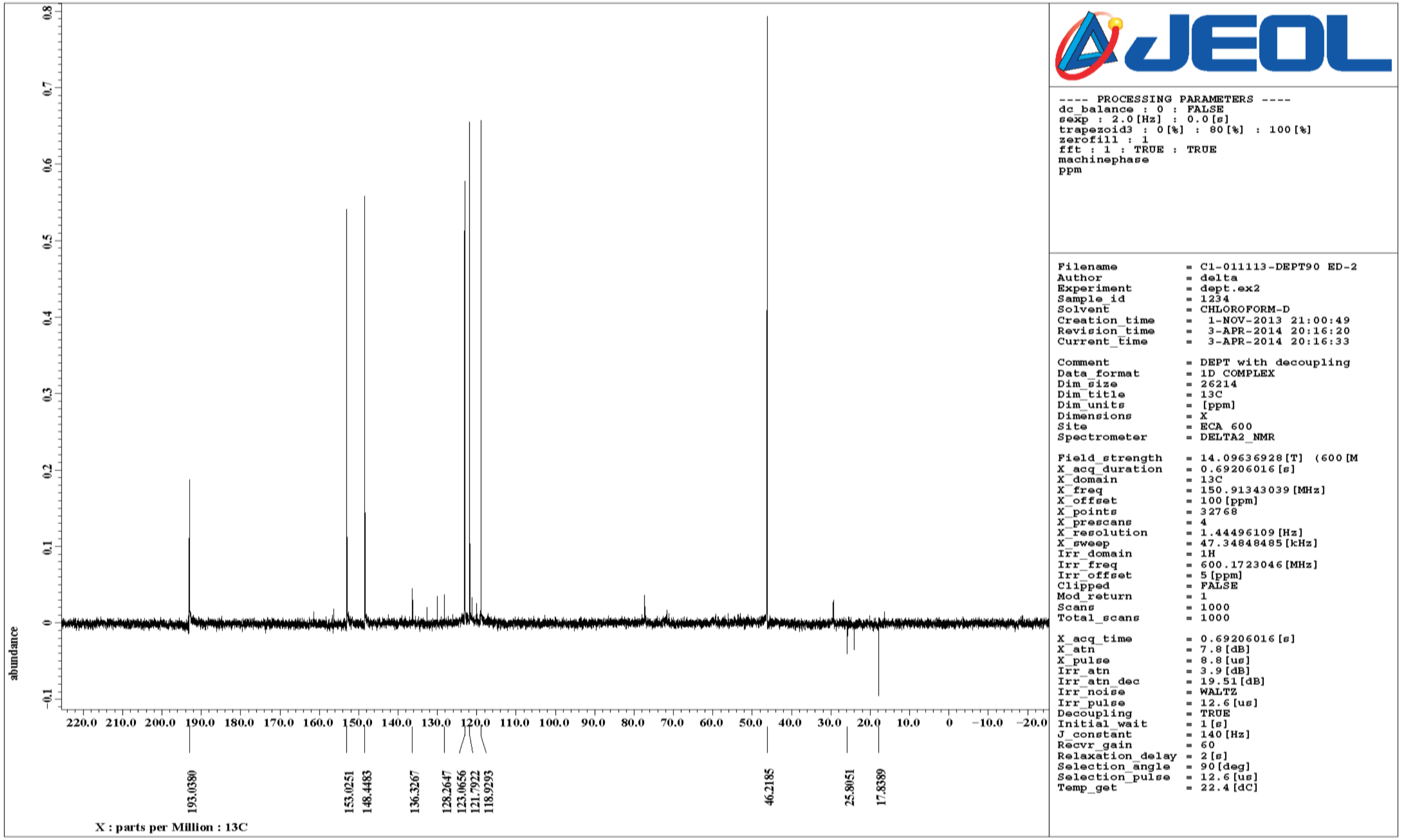

Figure S8. DEPT-135 NMR spectrum of cubelin.

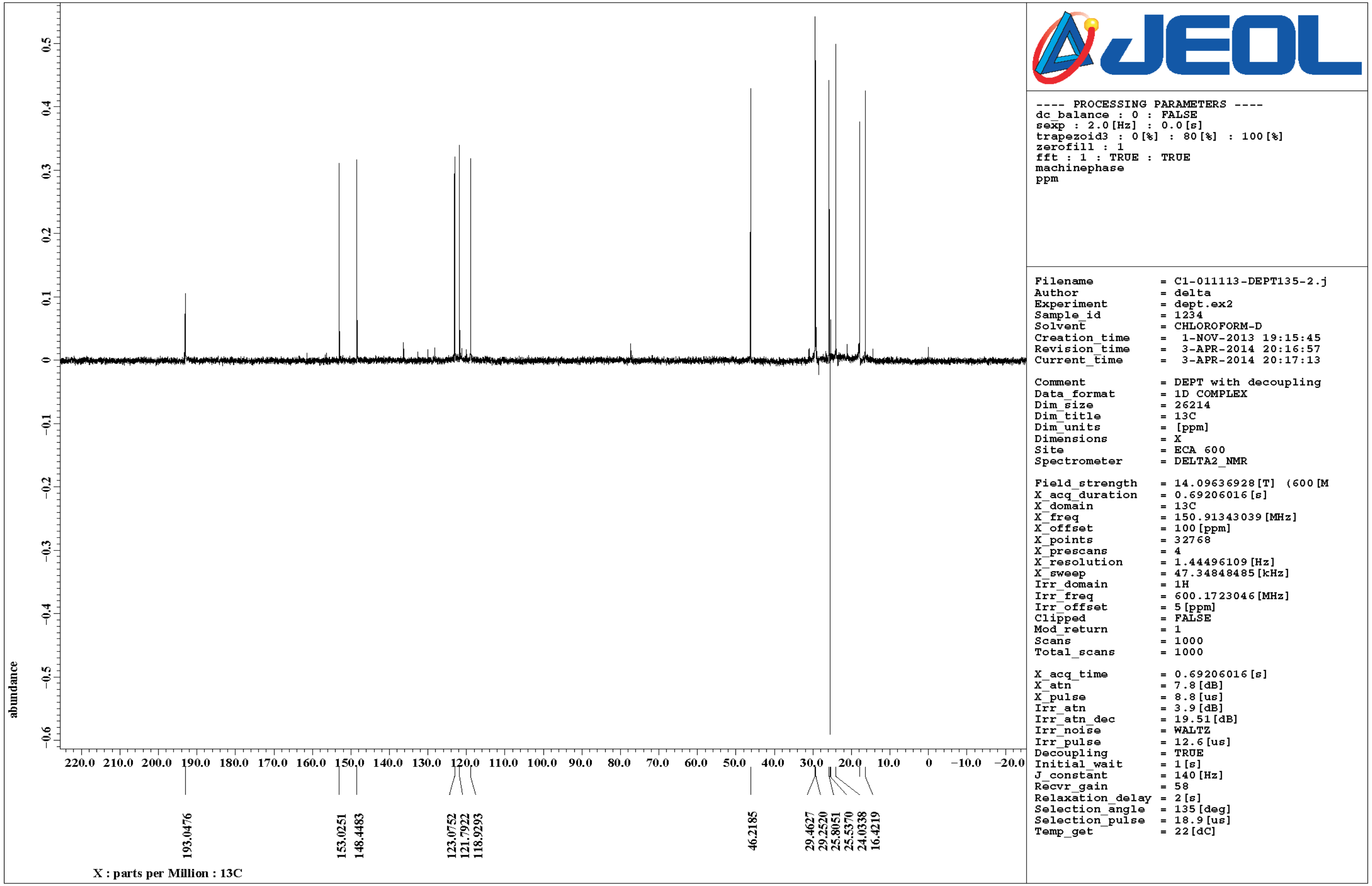

Figure S9. ^{1}H-^{1}H COSY NMR spectrum of cubelin

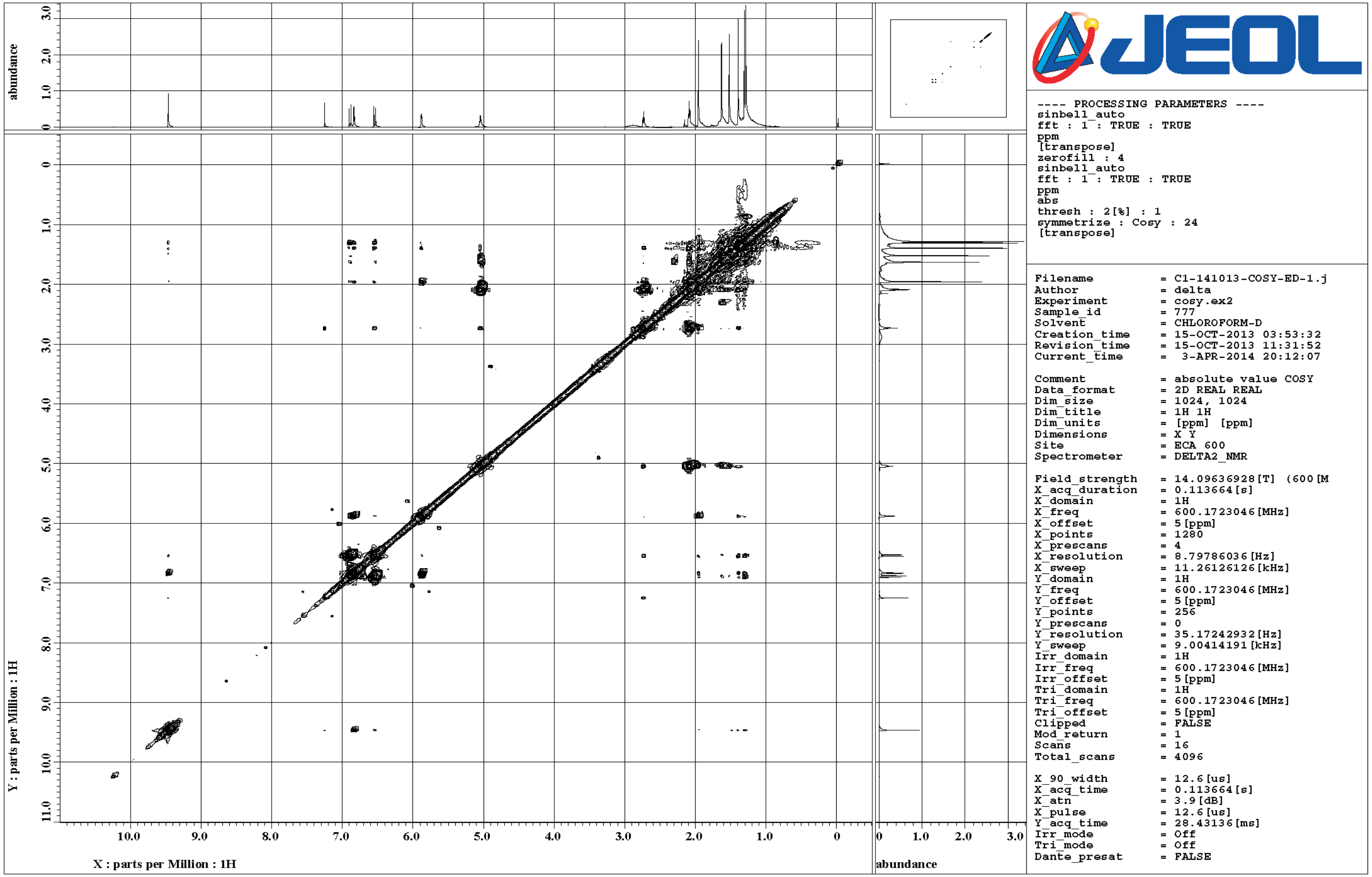

Figure S10. HETCOR NMR spectrum of cubelin

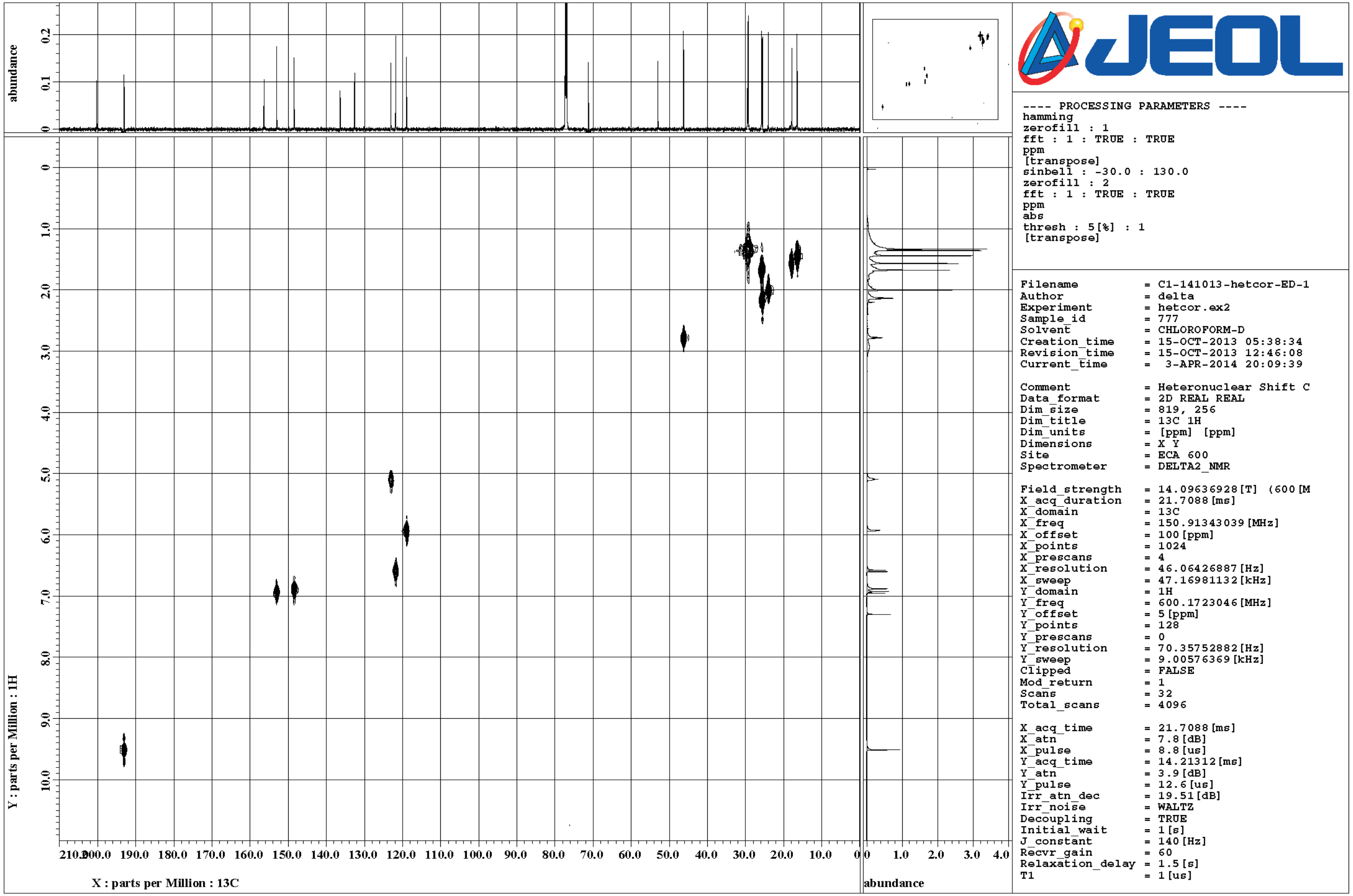

Figure S11. HMBC NMR spectrum of cubelin

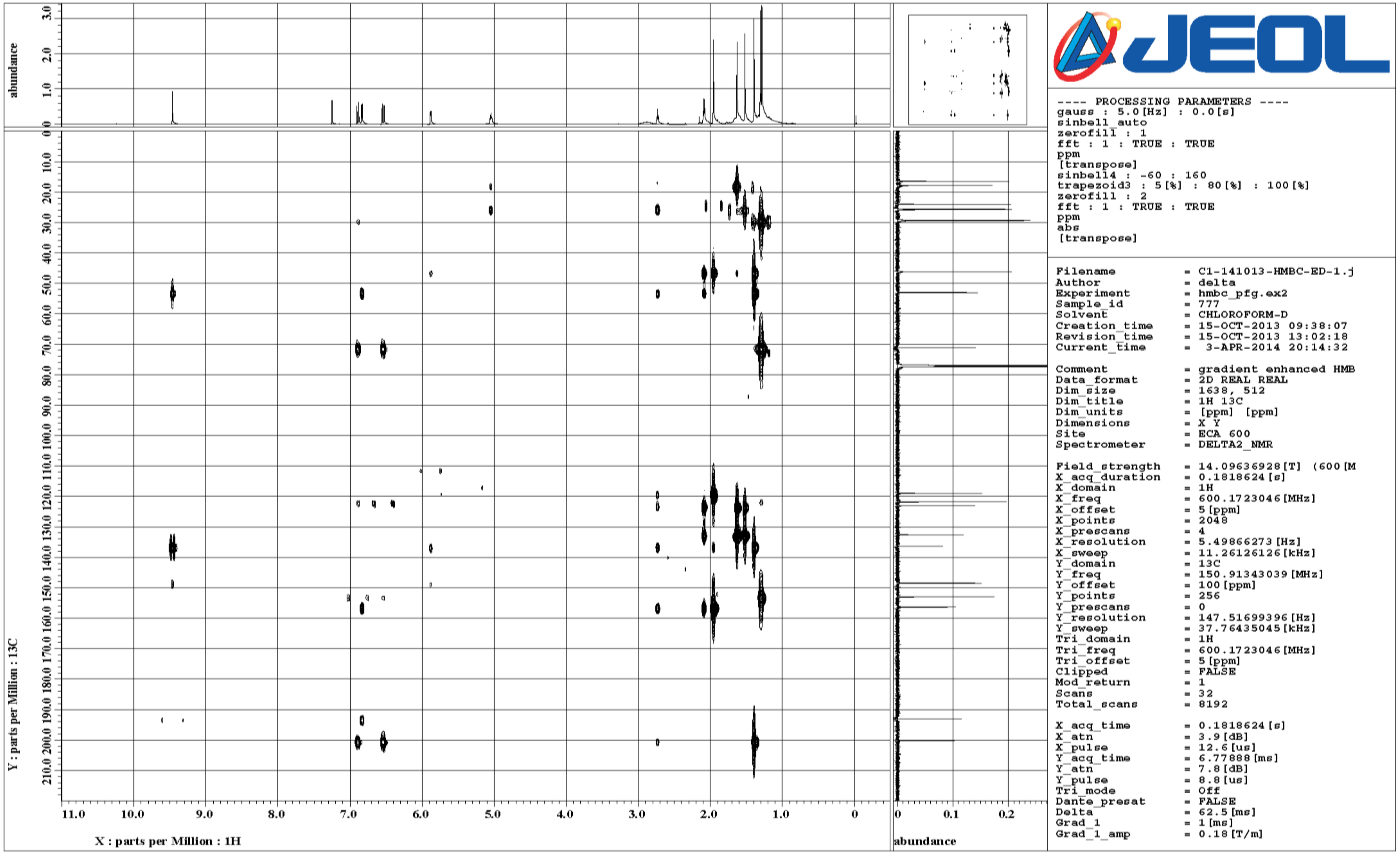

Figure S12. NOESY NMR spectrum of cubelin

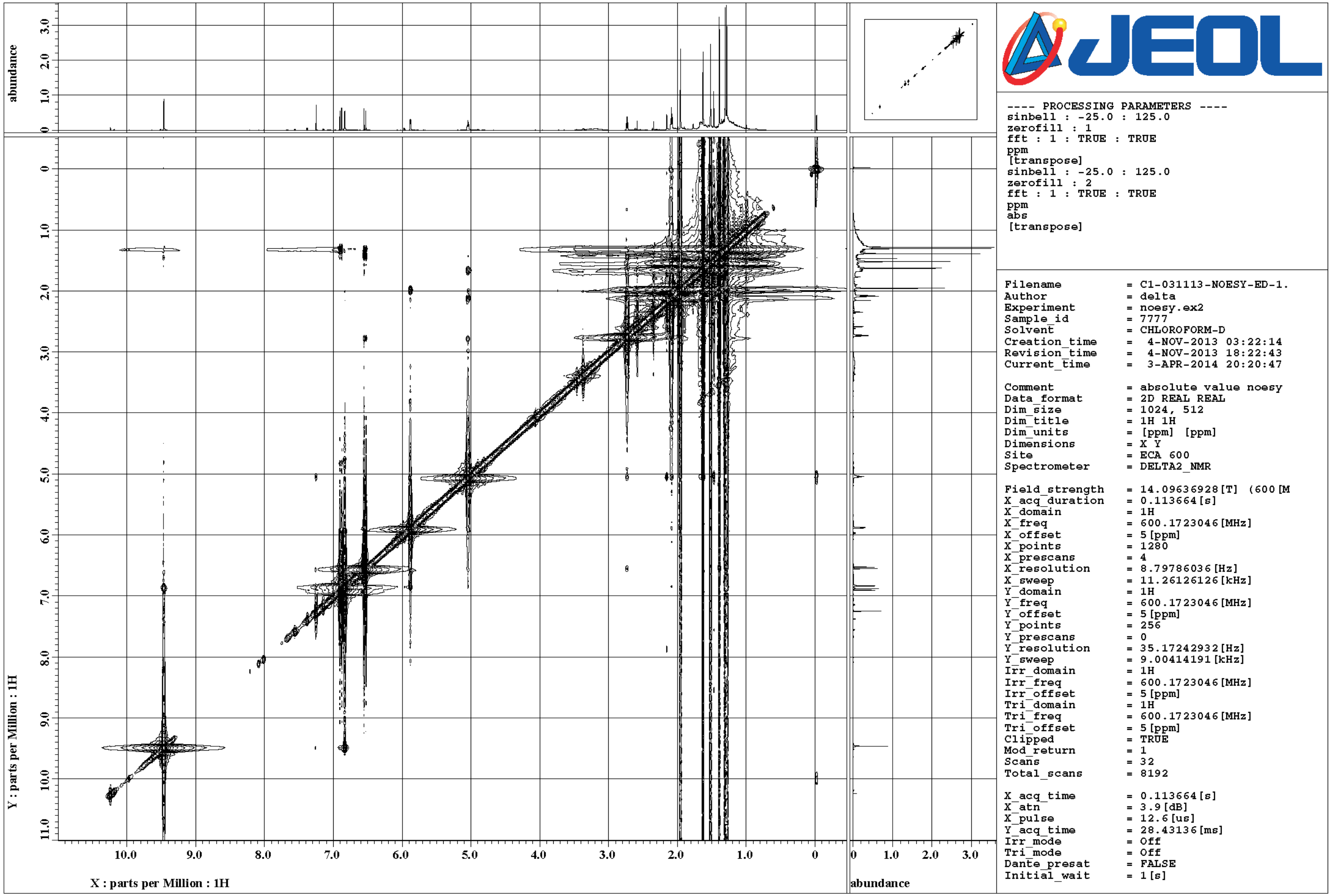

Figure S13. Specific Optical Rotation data of cubelin

```
P-1000 for Windows
Optical rotation measurement

Model : P-1030 (A010160639)
No.     Sample  Mode     Data    Monitor Temp.   HTV      Date    Blank    Cell length     Conc.   [Alpha] Sugar Coef      [Alpha]STD      Brix Conc.      Factor  Temp.(STD)      Temp Coef       Light   Filter  Cycle Time
Comment Sample Name      Operator        Temp Point
1        1 (1/10)        Specific O.R.    272.650         0.5453 20.2    406      Sat Dec 07 13:36:34 2013        0.0000 10.00   2.0000  0.000   17.313  66.450  1.00    1.0000  20.0    0.00000 Na      589nm   20 sec  5 sec
C1_Litsea       a        Holder
2        1 (2/10)        Specific O.R.    274.550         0.5491 20.1    386      Sat Dec 07 13:36:55 2013        0.0000 10.00   2.0000  0.000   17.313  66.450  1.00    1.0000  20.0    0.00000 Na      589nm   20 sec  5 sec
C1_Litsea       a        Holder
3        1 (3/10)        Specific O.R.    275.200         0.5504 20.1    410      Sat Dec 07 13:37:15 2013        0.0000 10.00   2.0000  0.000   17.313  66.450  1.00    1.0000  20.0    0.00000 Na      589nm   20 sec  5 sec
C1_Litsea       a        Holder
4        1 (4/10)        Specific O.R.    275.050         0.5501 20.1    390      Sat Dec 07 13:37:35 2013        0.0000 10.00   2.0000  0.000   17.313  66.450  1.00    1.0000  20.0    0.00000 Na      589nm   20 sec  5 sec
C1_Litsea       a        Holder
5        1 (5/10)        Specific O.R.    273.350         0.5467 20.1    390      Sat Dec 07 13:37:56 2013        0.0000 10.00   2.0000  0.000   17.313  66.450  1.00    1.0000  20.0    0.00000 Na      589nm   20 sec  5 sec
C1_Litsea       a        Holder
6        1 (6/10)        Specific O.R.    273.950         0.5479 20.1    401      Sat Dec 07 13:38:16 2013        0.0000 10.00   2.0000  0.000   17.313  66.450  1.00    1.0000  20.0    0.00000 Na      589nm   20 sec  5 sec
C1_Litsea       a        Holder
7        1 (7/10)        Specific O.R.    274.250         0.5485 20.2    382      Sat Dec 07 13:38:36 2013        0.0000 10.00   2.0000  0.000   17.313  66.450  1.00    1.0000  20.0    0.00000 Na      589nm   20 sec  5 sec
C1_Litsea       a        Holder
8        1 (8/10)        Specific O.R.    274.000         0.5480 20.2    405      Sat Dec 07 13:38:57 2013        0.0000 10.00   2.0000  0.000   17.313  66.450  1.00    1.0000  20.0    0.00000 Na      589nm   20 sec  5 sec
C1_Litsea       a        Holder
9        1 (9/10)        Specific O.R.    273.250         0.5465 20.1    406      Sat Dec 07 13:39:17 2013        0.0000 10.00   2.0000  0.000   17.313  66.450  1.00    1.0000  20.0    0.00000 Na      589nm   20 sec  5 sec
C1_Litsea       a        Holder
10       1 (10/10)       Specific O.R.    274.000         0.5480 20.1    398      Sat Dec 07 13:39:37 2013        0.0000 10.00   2.0000  0.000   17.313  66.450  1.00    1.0000  20.0    0.00000 Na      589nm   20 sec  5 sec
C1_Litsea       a        Holder
11       2 (1/10)        Specific O.R.    277.600         0.2776 20.1    390      Sat Dec 07 13:44:29 2013        0.0000 10.00   1.0000  0.000   17.313  66.450  1.00    1.0000  20.0    0.00000 Na      589nm   20 sec  5 sec
C1_Litsea       a        Holder
12       2 (2/10)        Specific O.R.    279.200         0.2792 20.2    375      Sat Dec 07 13:44:49 2013        0.0000 10.00   1.0000  0.000   17.313  66.450  1.00    1.0000  20.0    0.00000 Na      589nm   20 sec  5 sec
C1_Litsea       a        Holder
13       2 (3/10)        Specific O.R.    276.300         0.2763 20.2    382      Sat Dec 07 13:45:10 2013        0.0000 10.00   1.0000  0.000   17.313  66.450  1.00    1.0000  20.0    0.00000 Na      589nm   20 sec  5 sec
C1_Litsea       a        Holder
14       2 (4/10)        Specific O.R.    272.400         0.2724 20.1    398      Sat Dec 07 13:45:30 2013        0.0000 10.00   1.0000  0.000   17.313  66.450  1.00    1.0000  20.0    0.00000 Na      589nm   20 sec  5 sec
C1_Litsea       a        Holder
15       2 (5/10)        Specific O.R.    274.500         0.2745 20.1    382      Sat Dec 07 13:45:50 2013        0.0000 10.00   1.0000  0.000   17.313  66.450  1.00    1.0000  20.0    0.00000 Na      589nm   20 sec  5 sec
C1_Litsea       a        Holder
16       2 (6/10)        Specific O.R.    275.700         0.2757 20.1    378      Sat Dec 07 13:46:11 2013        0.0000 10.00   1.0000  0.000   17.313  66.450  1.00    1.0000  20.0    0.00000 Na      589nm   20 sec  5 sec
C1_Litsea       a        Holder
17       2 (7/10)        Specific O.R.    275.800         0.2758 20.1    404      Sat Dec 07 13:46:31 2013        0.0000 10.00   1.0000  0.000   17.313  66.450  1.00    1.0000  20.0    0.00000 Na      589nm   20 sec  5 sec
C1_Litsea       a        Holder
18       2 (8/10)        Specific O.R.    276.500         0.2765 20.2    374      Sat Dec 07 13:46:51 2013        0.0000 10.00   1.0000  0.000   17.313  66.450  1.00    1.0000  20.0    0.00000 Na      589nm   20 sec  5 sec
C1_Litsea       a        Holder
19       2 (9/10)        Specific O.R.    278.800         0.2788 20.2    405      Sat Dec 07 13:47:12 2013        0.0000 10.00   1.0000  0.000   17.313  66.450  1.00    1.0000  20.0    0.00000 Na      589nm   20 sec  5 sec
C1_Litsea       a        Holder
20       2 (10/10)       Specific O.R.    277.400         0.2774 20.2    378      Sat Dec 07 13:47:32 2013        0.0000 10.00   1.0000  0.000   17.313  66.450  1.00    1.0000  20.0    0.00000 Na      589nm   20 sec  5 sec
C1_Litsea       a        Holder
```
